# Diagnostic pitfall of thyroid fine-needle aspiration induced fibrosis: follicular adenoma mimicking medullary thyroid carcinoma in frozen section

**DOI:** 10.1186/s13000-021-01087-2

**Published:** 2021-03-17

**Authors:** Woo Sung Moon, Myoung Jae Kang, Hyun Jo Youn, Kyoung Min Kim

**Affiliations:** 1 Department of Pathology, Jeonbuk National University Medical School, Jeonbuk National University Medical School, 567 Baekje-daero, Doekjin-gu, Jeonju-si, Jeollabuk-do 561-756 Republic of Korea; 2 Division of Breast Thyroid Surgery, Department of Surgery, Jeonbuk National University Medical School, Jeonju, Republic of Korea; 3 Research Institute of Clinical Medicine of Jeonbuk National University-Biomedical Research Institute of Jeonbuk National University Hospital, Jeonju, Republic of Korea

**Keywords:** Frozen section, Fine-needle aspiration, Fibrosis, Follicular adenoma, Medullary thyroid carcinoma, Case report

## Abstract

**Background:**

Fine-needle aspiration (FNA) is a frequently utilized method for the diagnosis of thyroid nodules. Although the technique has clear advantages, the injury caused by the aspiration needle can induce various histological alterations. Herein, we report a case of follicular adenoma showing histological alterations possibly caused by FNA biopsy. Furthermore, the histological appearance of the lesion mimicked those of medullary thyroid carcinoma, particularly in the frozen section.

**Case presentation:**

Ultrasonography of a thyroid nodule in a 39-year-old man revealed a mass (2.2 cm in diameter) in the right thyroid lobe. FNA was performed three times on the mass, and the results of the cytology were atypia of undetermined significance. Thereafter, the patient underwent right hemithyroidectomy. The histological findings of the operative frozen section analysis indicated medullary thyroid carcinoma. However, after evaluation and immunohistochemical staining of the permanent section, the mass was diagnosed as follicular adenoma with extensive fibrosis.

**Conclusion:**

The histological alterations observed in the follicular adenoma are believed to have been caused by injury during the repeated FNA procedures.

## Introduction

Fine-needle aspiration (FNA) is a widely recommended diagnostic method for the initial evaluation of a thyroid nodule [1, 2]. This technique has been proven to be quick, safe, and well-tolerated with minimal complications [1]. However, despite the clear advantages of this method as a diagnostic tool, injuries induced by the aspiration needle can alter the histology of the tissues leading to diagnostic misinterpretations [3]. Herein, we report a case of follicular adenoma with extensive fibrosis, possibly caused by FNA, mimicking medullary thyroid carcinoma.

## Case report

**Fig. 1:**
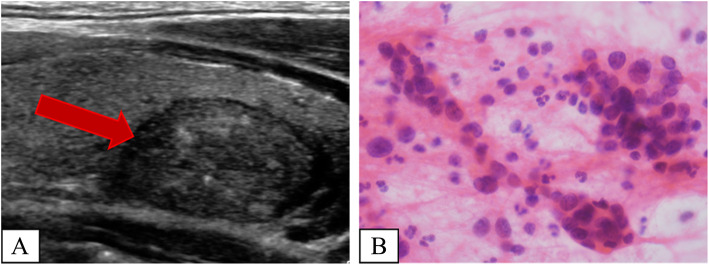
Ultrasonographic and cytologic features of the thyroid mass. **a** Ultrasonography reveals a relatively well-circumscribed thyroid mass on the right lobe (arrow). **b** Some follicular cells showing nuclear enlargement and pale chromatin pattern in cytologic evaluation of FNA specimen (H&E stain; original magnification, × 1000)

A 39-year-old man presented at our hospital with a thyroid nodule in the neck region, which the mass had been detected during a routine medical check-up. FNA were performed twice prior to the visit to our hospital, but the results of the cytological evaluations at both instances were atypia of undetermined significance (AUS). The patient had no clinical symptoms and the laboratory results were in normal range. Ultrasonography, performed at our hospital, revealed the presence of a relatively well circumscribed mass (2.2 cm in diameter) with microcalcifications on the right lobe of the thyroid gland (Fig. 1a). Additionally, another FNA conducted at our hospital. The cytologic evaluation showed some follicular cells with enlarged nuclei and pale chromatin pattern. However, the cytologic findings were not sufficient to diagnose a specific tumor, thereafter we diagnosed the cytologic specimen as AUS (Fig. 1b). Subsequently, the patient underwent right hemithyroidectomy for diagnostic and treatment purposes.

**Fig. 2:**
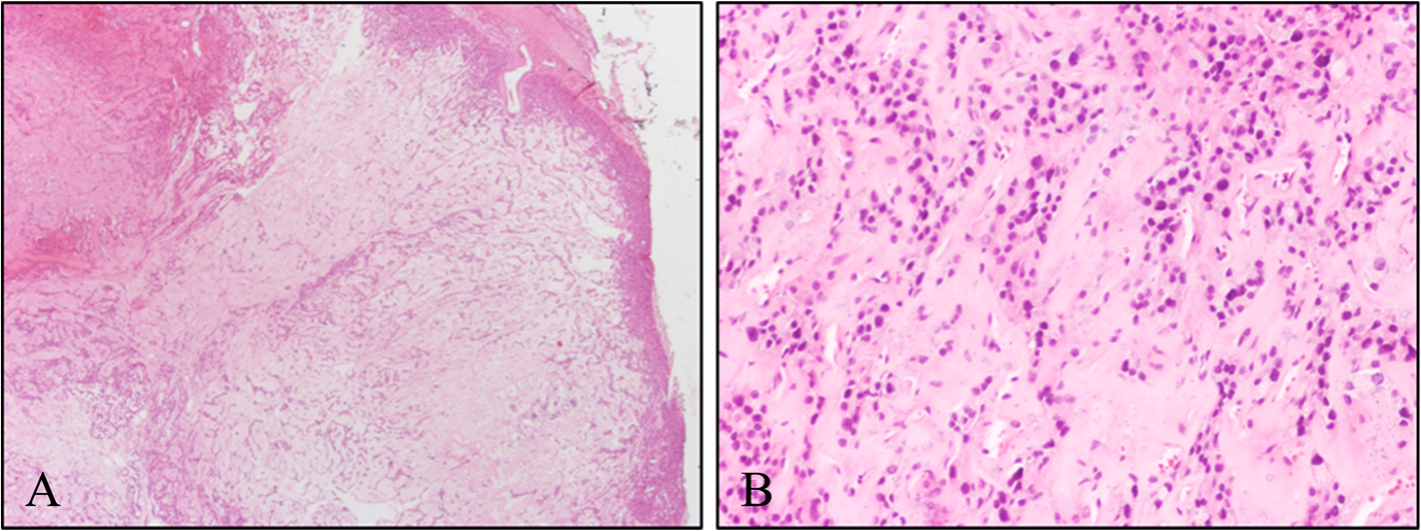
Hematoxylin and eosin (H&E)-stained frozen section of the thyroid mass. **a** At low magnification (× 20), the tumor was relatively well-circumscribed. **b** At high magnification (× 200), the tumor cells were found to be arranged in a trabecular pattern between homogenous eosinophilic materials

The results of the cytology could not confirm whether the mass was benign or malignant. Hence, histologic evaluation of a frozen section using hematoxylin-eosin (H&E) staining was performed. A relatively well-circumscribed mass comprising cells that were mostly arranged in a trabecular or solid sheet-like pattern and intersected by homogenous eosinophilic material was noted (Fig. 2). The tumor cells consisted of amphophilic cytoplasm and round to oval-shaped nuclei with condensed chromatin and indistinct nucleoli (Fig. 2b). The eosinophilic material was suspected to be amyloid deposition and the mass was diagnosed as a medullary thyroid carcinoma. Neck lymph node dissection was additionally performed due to the results of the frozen biopsy. And the patient has been warned that if the results of permanent biopsy was confirmed to medullary thyroid carcinoma as of the frozen biopsy, total thyroidectomy is necessary.

**Fig. 3:**
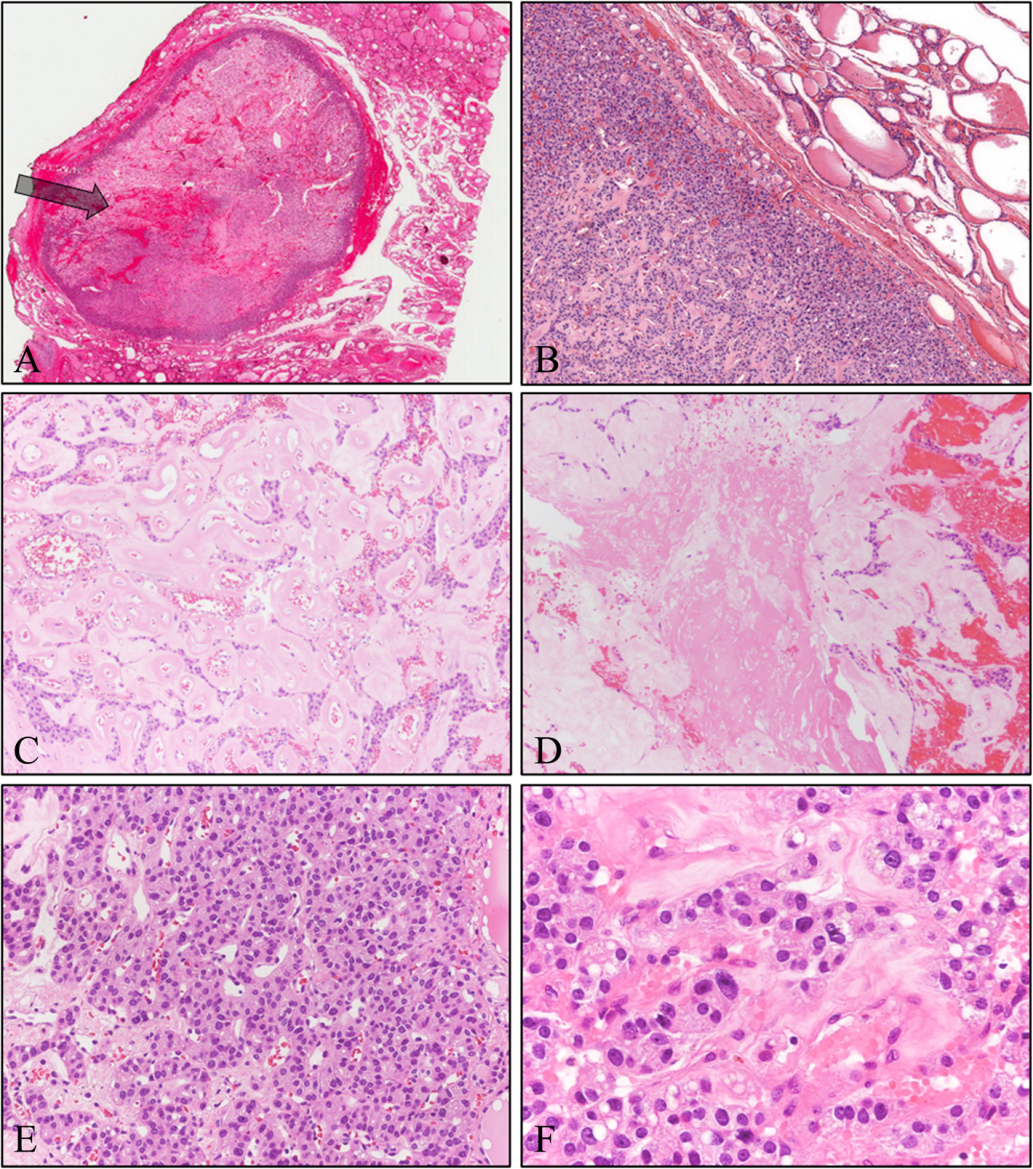
Histological features of the mass observed from a permanent section. **a** At scan view, the tumor shows well-margination and needle tract-like structure (arrow) (H&E stain; original magnification, scan view). **b** The tumor showing thin but obvious fibrous capsule (H&E stain; original magnification, × 40). **c** The tumor cells showed trabecular growth patterns with intersecting homogenous eosinophilic materials (H&E stain; original magnification, × 100). **d** Infarction-like necrotic area and hemorrhage is present in some areas of the tumor (H&E stain; original magnification, × 100). **f** The tumor cells showed amphophilic granular cytoplasm with round to oval nuclei and nucleoli in some cells (H&E stain; original magnification, × 400)

**Fig. 4:**
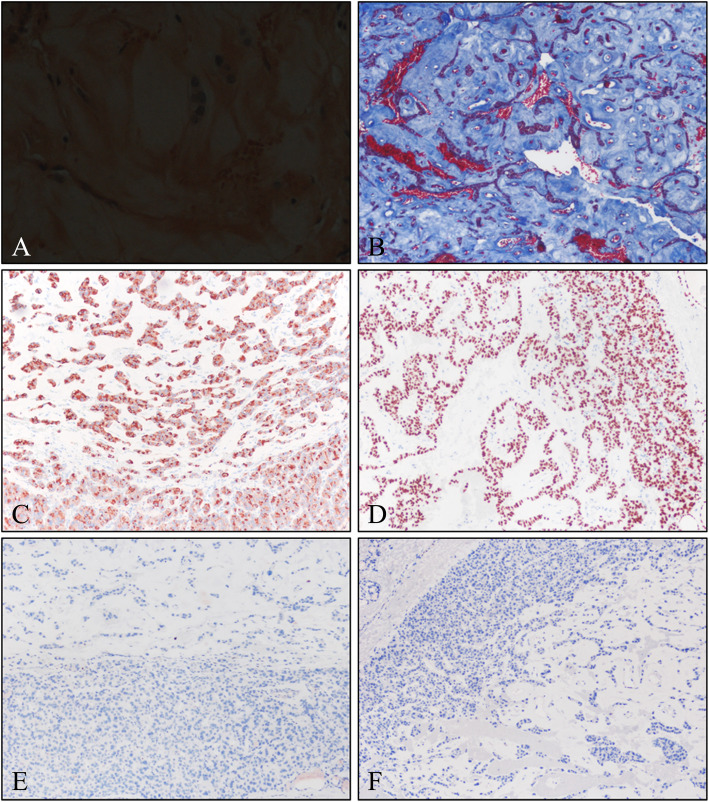
Special stain and immunohistochemical features of the mass. **a** Congo Red staining of the section did not reveal any apple-green birefringence under polarized light microscopy (original magnification, × 400). **b** The homogenous eosinophilic materials stained blue with Masson trichrome stain (original magnification, × 100). The tumor cells showing immunoreactivity for thyroglobulin (**c**); original magnification, × 100) and TTF-1 (**d**); original magnification, × 100). The tumor cells did not express immunoreactivity for synaptophysin (**e**); original magnification, × 100) or calcitonin (**f**); original magnification, × 100)

Subsequently, a permanent section obtained from the patient was evaluated. The tumor showed well margination with thin fibrous capsule (Fig. 3a and b). The basic growth pattern and morphology of the tumor cells were similar to those seen in the frozen section (Fig. 3c). Additionally, follicular growth pattern was identified in the permanent section (Fig. 3e). Also, infarction-like necrotic area and hemorrhage were recognized (Fig. 3d). Congo Red staining of the homogenous eosinophilic material failed to show the characteristic apple-green birefringence under polarized light microscopy (Fig. 4a). And blue staining of the homogenous material using Masson’s trichrome staining method confirmed that the homogenous eosinophilic material was fibrosis (Fig. 4b). Immunohistochemical staining showed diffuse immunopositivity for thyroglobulin and thyroid transcription factor-1 (TTF-1) (Fig. 4c and d, respectively). Conversely, no staining for synaptophysin or calcitonin, known to be expressed in medullary thyroid carcinoma, was observed (Fig. 4e and f, respectively). Knowing the patient’s history of multiple FNA procedures during the diagnosis, we suspected that the fibrosis, hemorrhage and necrosis was due to the injury from the FNA. Additionally, there was a needle tract-like structure in low-power field (Fig. 3a, arrow). Therefore, based on above findings the mass was diagnosed as follicular adenoma with extensive fibrosis. The patient is alive without recurrence or metastasis, after 3 years of follow up.

## Discussion

The incidence of thyroid cancer has been rapidly increasing since the mid-1990s [1]. FNA is the first choice for diagnosing thyroid lesions because of the high sensitivity and specificity of this method [1]. Additionally, it is believed to be safe and associated with fewer complications when compared with other methods; one of the main complications of FNA is the occurrence of minor hematomas [4]. Thus, for these reasons, the usage of the FNA method has increased dramatically over the years.

However, this method has some limitations. A definitive cytological diagnosis cannot be made in 10 to 25% of the thyroid FNA cases [5]. One of the main reasons for this is the presence of atypical cells for which the significance cannot be determined [5]. The recently updated Bethesda guidelines for the management of thyroid nodules classifies cases harboring atypical cells that cannot be determined as AUS [6]. Furthermore, they recommend repeat FNA for a more conclusive result because the reported risk for malignancy in cases with AUS is 5–15% [6]. The number of patients requiring repeat FNA has increased owing to the rapid increase in the incidence of thyroid FNA biopsies. The patient in the current case report underwent repeated FNA because the thyroid nodule was cytologically diagnosed as AUS.

Although the FNA method is very effective and accurate for the diagnosis of thyroid lesions, trauma caused by the aspiration needle can induce varying degrees of histological alterations [3]. These alterations induced by FNA have been classified into acute and chronic types based on the type of histological alteration and the interval between the FNA procedure and the surgery [7]. Acute alterations include hemorrhage, granulation tissue formation, siderophagia, necrosis, granulomatous inflammation, and associated cytological atypia. The most common findings are hemorrhage and granulation tissue formation, which are predominantly found with a 3-week interval between the FNA procedure and the surgery [7]. On the other hand, chronic alterations include fibrosis, metaplasia, infarction, capsular distortion, cyst formation, papillary degeneration, papillary endothelial proliferation, and calcification [7]; fibrosis and distortion of the capsule are most frequently observed in this group with an interval period of 3 weeks to 6 months [7].

For pathologists, capsular and vascular alteration due to FNA procedure has caused difficulties to diagnosis [5]. Because capsular or vascular invasion is the most important factor for differentiating follicular adenoma from follicular carcinoma [5]. In our present case, we have embedded the entire capsule of the mass and evaluated the capsular invasion. Fortunately, the fibrous capsule of our case was intact and therefore follicular carcinoma could be ruled out. Reactive atypia due to previous FNA procedure can also be a problem in diagnosis of thyroid neoplasm [5]. Reactive cytologic atypia, such as nuclear clearing, mitosis, prominent nucleoli, and nuclear pleomorphism can occur in stromal and follicular cells following FNA procedure. And these cytologic features can create a confusion between papillary thyroid carcinoma, which is the most prevalent malignant tumor in thyroid gland, and benign thyroid lesion with FNA induced reactive cytologic atypia [5]. However, in the present case, fibrosis caused diagnostic difficulties while evaluating thyroid lesions. Although, follicular adenomas can exhibit various secondary changes including fibrosis as in our case, we suspected that the fibrosis was induced by FNA procedure because in addition to fibrosis, hemorrhage, infarction type necrosis and needle tract-like structure (Fig. 2a, arrow) were identified and most of all the patient’s history of multiple FNA procedure before the surgery. Fibrosis is one of the most common FNA-induced chronic alterations [8]. It is a common reactive reaction that affects most thyroid lesions, benign or malignant [4]. In the study by Bolat F, fibrosis was identified in 66.0% patients who underwent FNA, whereas only 15.3% of the cases without a history of FNA presented with fibrosis [8].

In our present case, the fibrosis created significant challenges in distinguishing between follicular adenoma and medullary carcinoma. The tumor cells in medullary thyroid carcinoma are usually arranged in nests or trabecular patterns separated by varying amounts of fibrovascular stroma [9]. The tumor stroma appears variable; however, abundant hyalinized collagen and Congo Red-positive stromal amyloid deposits have been observed in 80% of medullary thyroid carcinoma cases [9].

Distinguishing medullary thyroid carcinoma from follicular adenoma with fibrosis is relatively simple because the tumor cells of the carcinoma show immunoreactivity for calcitonin and neuroendocrine markers, such as chromogranin A and synaptophysin. However, in the present case, we had to examine frozen sections without immunohistochemistry, making it extremely difficult to reach an accurate diagnosis and consequently leading to a misdiagnosis. In frozen sections, the tumor cells were arranged in a trabecular pattern due to fibrosis and the thick collagenous tissues mimicked amyloid deposition similar to that seen in medullary thyroid carcinoma. These findings indicate the importance of ascertaining the medical history of the patient, with particular emphasis on the number of FNA procedures undergone, before diagnosing thyroid nodules.

Historically, intraoperative frozen biopsy has been utilized to guide the surgical treatment strategy of thyroid nodule [10]. If the frozen biopsy diagnosis is benign, surgical treatment could be sufficient with a hemi-thyroidectomy, while a total thyroidectomy should be done in case of a malignant tumor [10]. The role of frozen biopsy has been controversy since the emergence of FNA as the main preoperative diagnostic tool of thyroid nodules [10]. However, as mentioned above, there are lesions that cannot be determined by cytology alone and diagnosed as AUS. And for these indeterminate thyroid nodules, frozen biopsy is still regarded as a useful tool for deciding the strategy of surgery. In previous study, the frozen biopsy accuracy was reported to be 78.9%, higher than that of FNA [11]. And the frozen biopsy had above 90% specificity in many other studies [12–15]. In the present case, repeated cytologic diagnosis of FNA were AUS, and therefore the surgeons have requested frozen biopsy to determine the extent of surgery. As mentioned above, it was very difficult to distinguish follicular adenoma with extensive fibrosis from medullary thyroid carcinoma in frozen biopsy, especially due to the fibrosis mimicking amyloid deposition. Most cases of medullary thyroid carcinomas show infiltrative borders with infiltration of the tumor cells into the surrounding normal tissue [9]. In contrast, follicular adenoma has an intact capsule, although the FNA procedure can damage the capsule. In the current case report, the capsule was intact. Therefore, evaluating the status of the capsule could be one of the distinctive points for differentiating follicular adenoma with fibrosis from medullary thyroid carcinoma for pathologist when immunohistochemistry cannot be used.

In conclusion, we have presented a case of follicular adenoma with extensive fibrosis with features mimicking those of medullary thyroid carcinoma caused by FNA biopsy. It is important that the pathologist be aware of FNA-induced histopathological alterations in order to avoid the misdiagnosis of thyroid nodules and lead to unnecessary treatment.

## Data Availability

As a case report, all data generated or analyzed are included in this article.

## Abbreviations

FNA: Fine-needle aspiration
AUS: Atypia of undetermined significance
TTF-1: Thyroid transcription factor-1
